# Metabolomics dataset of mouse optogenetic axon regeneration after optic nerve crush

**DOI:** 10.1016/j.dib.2022.108306

**Published:** 2022-05-21

**Authors:** Alexa M. Jauregui, Yuan Liu, Sanjoy K. Bhattacharya, Richard K. Lee

**Affiliations:** a Bascom Palmer Eye Institute, University of Miami Miller School of Medicine, Miami, FL, 33136, USA; b Miami Integrative Metabolomics Research Center, Miami, FL, 33136, USA; c University of Miami Miller School of Medicine, Miami, FL, 33136, USA

**Keywords:** Optic nerve crush, Optogenetics, Axon regeneration, Metabolomics, High-performance liquid chromatography

## Abstract

This metabolite dataset was collected from transgenic murine retinal ganglion cells (RGC) expressing bacterial channelrhodopsin labeled with fluorescent protein. Mice were subjected to optic nerve crush (ONC) with subsequent RGC stimulation of channelrhodopsin with blue light (promoting regeneration) or non-stimulation (control). ONC induces retinal ganglion cell degeneration over time with progressive loss of axons. In transgenic bacterial channelrhodopsin expressing RGC cells, light stimulation promotes regeneration of ONC axons. Genetically matched wild-type uninjured optic nerves were analyzed as controls for comparison.

Metabolites were carefully extracted from finely minced optic nerve tissue using a solvent system (initial separation using 1:1 methanol and H_2_O and second extraction using 8:1:1 of acetonitrile:acetone:methanol). Untargeted liquid chromatography-mass spectrometry profiling was performed using fractionation on a Vanquish Horizon Binary UHPLC. Subsequent analyses were performed on an inline coupled Q-Exactive Orbitrap instrument. Metabolites were identified using Compound Discoverer^TM^ software. Statistical analysis was performed using MetaboAnalyst 5.0. This data is available on Metabolomics Workbench, Study ID ST002111.

## Specifications Table

**Table:** 

Subject	Ophthalmology
Specific subject area	Metabolites in neuronal regeneration
Type of data	Chart Chromatogram Figure Graph Spectra
How the data were acquired	High-Performance Liquid Chromatography, Q Exactive Orbitrap Mass Spectrometer
Data format	Raw Analyzed Filtered
Description of data collection	Optic nerves were collected from channelrhodopsin (Thy1-ChR2-eYFP) and control (C57BL/6J) mice eyes. Thy1-ChR2-eYFP and C57BL/6J were divided into five and three groups, respectively, for a total of eight conditions for comparison. Optic nerves from three mice were pooled per condition prior to metabolite extraction. Untargeted metabolomics was performed and analyzed using high-performance liquid chromatography and mass spectrometry.
Data source location	Bascom Palmer Eye Institute, Miller School of Medicine, University of Miami, Miami, FL 33136, USA
Data accessibility	This study is now available on Metabolomics Workbench, https://www.metabolomicsworkbench.org where it has been assigned Study ID ST002111. The data can be accessed directly via its Project DOI: http://dx.doi.org/10.21228/M8WM52.

## Value of the Data

• This data provides valuable insight regarding the metabolite changes which occur after traumatic optic nerve injury in Thy1-ChR2-eYFP mice and C57BL/6J control mice eyes.
• This data is beneficial for understanding the metabolic changes associated with axonal regeneration.
• This dataset provides insights into the regulatory changes that could be used for successful therapeutic treatments.
• The data can also be used to create metabolite spectral libraries and generate MS/MS data needed for future targeted metabolomic studies.

## 1 Data Description

We present a metabolomic contour and analyze the profile in Thy1-ChR2-eYFP mice and C57BL/6J mice RGCs. While a lipidomics experiment was previously performed using the same model [1], this is the first quantitative contour map of metabolites compared in the context of neuron regeneration in the optic nerve.

**Fig. 1:**
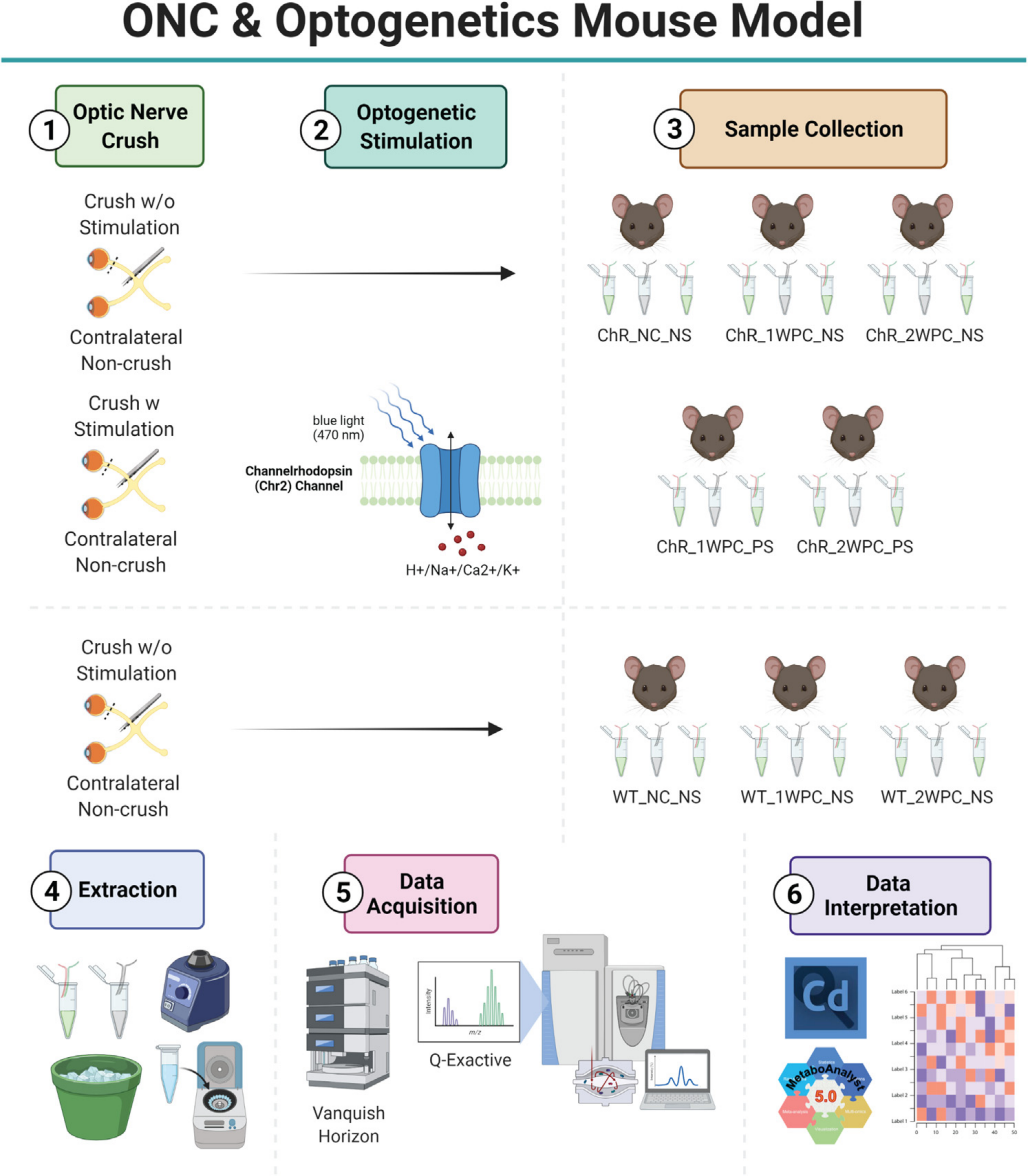
**Schematic representation of the metabolite profiling protocol used for the 1) optic nerve crush (ONC) and 2) optogenetics-induced mouse models: Thy1-Ch**R**2-eYFP mice and C57BL/6J mice.** All experimental samples were subjected to ONC. 2) The effects of light stimulation post-ONC were then compared at one week and two weeks post injury (ChR_1WPC_PS and ChR_2WPC_PS) in the channelrhodopsin model. 3) Data was collected for the following experimental groups for comparison: wild-type and channelrhodopsin mice without the effects of crush and light stimulation (WT_NC_NS and ChR_NC_NS). The effects of no stimulation after ONC at one week (WT_1WPC_NS and ChR_1WPC_NS) and two weeks (WT_2WPC_NS and ChR_2WPC_NS) in the absence and presence of channelrhodopsin. 4) A metabolite extraction was performed, and samples were subject to 5) untargeted liquid chromatography-mass spectrometry profiling. 6) Metabolites were identified and analyzed using Compound Discoverer 3.3. Data analysis and further stratification was performed using MetaboAnalyst 5.0.

The experimental groups and protocol followed are shown in Fig. 1. Wild-type and channelrhodopsin mice without the effects of crush and light stimulation (WT_NC_NS and ChR_NC_NS) were used as controls. The effects of no stimulation after optic nerve crush (ONC) were compared at one week (WT_1WPC_NS and ChR_1WPC_NS) and two weeks (WT_2WPC_NS and ChR_2WPC_NS) in the absence and presence of channelrhodopsin. The effects of light stimulation on RGCs post-ONC were compared at one week and two weeks post injury (ChR_1WPC_PS and ChR_2WPC_PS) in the channelrhodopsin model. Due to the small tissue size of a murine optic nerve, optic nerves were pooled from three mice per condition prior to performing the metabolite extraction.

**Fig. 2:**
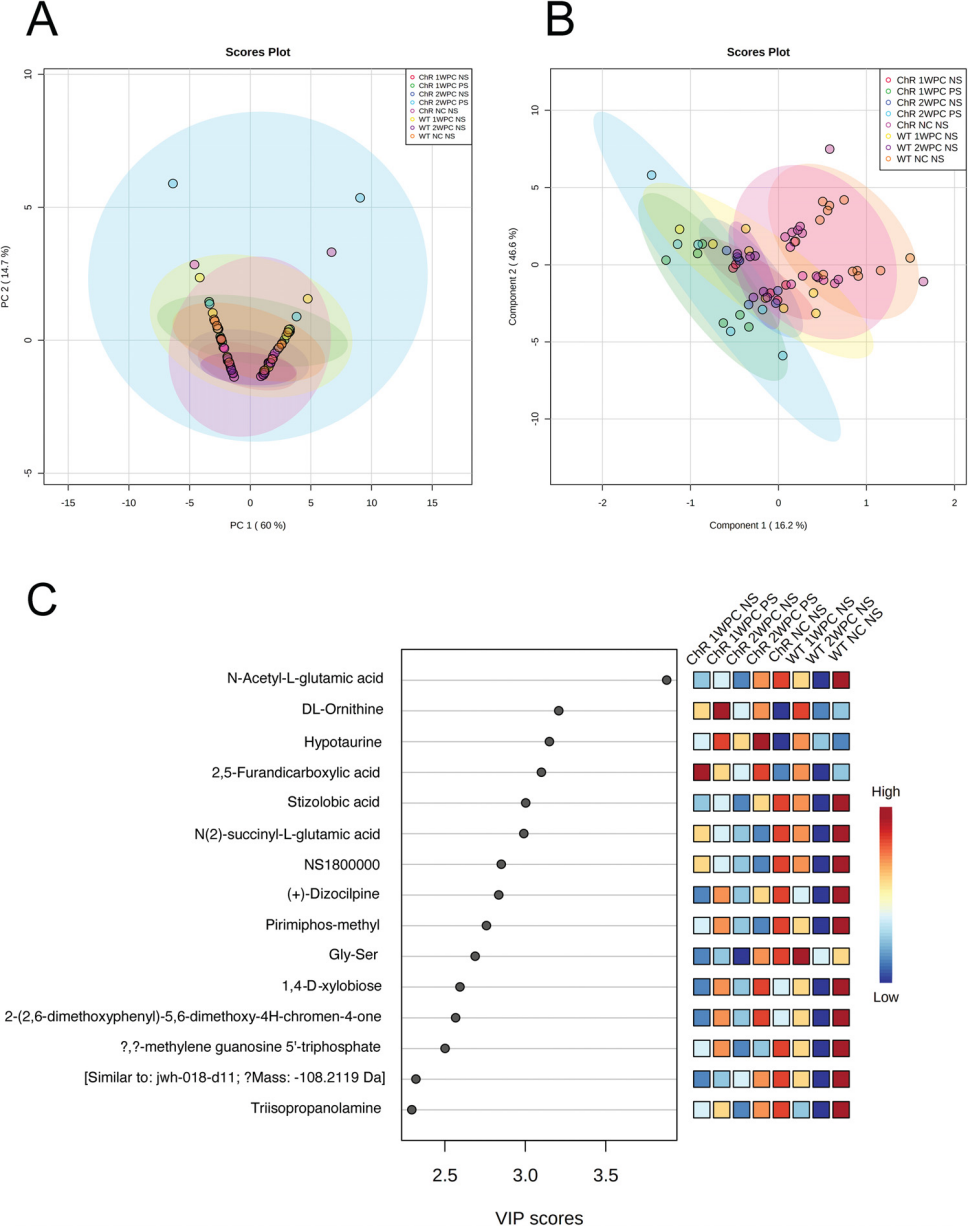
**Multivariate analysis of metabolite species identified in all experimental conditions of the ONC and optogenetics mouse model.** A) PCA 2-D scores plot of metabolite profiling data. The top 15 important features identified by PLS-DA and VIP scores are presented (B-C). The colored boxes on the right indicate the relative concentrations of the corresponding metabolite in each group under study. Red indicates a high concentration and blue indicates low concentration. **Abbreviations:** WT = wild-type (C57BL/6J mice), ChR = channelrhodopsin transgenic model, NC = no optic nerve crush, PC = post-optic nerve crush, PS = post-light stimulation NS = no light stimulation.

**Fig. 3:**
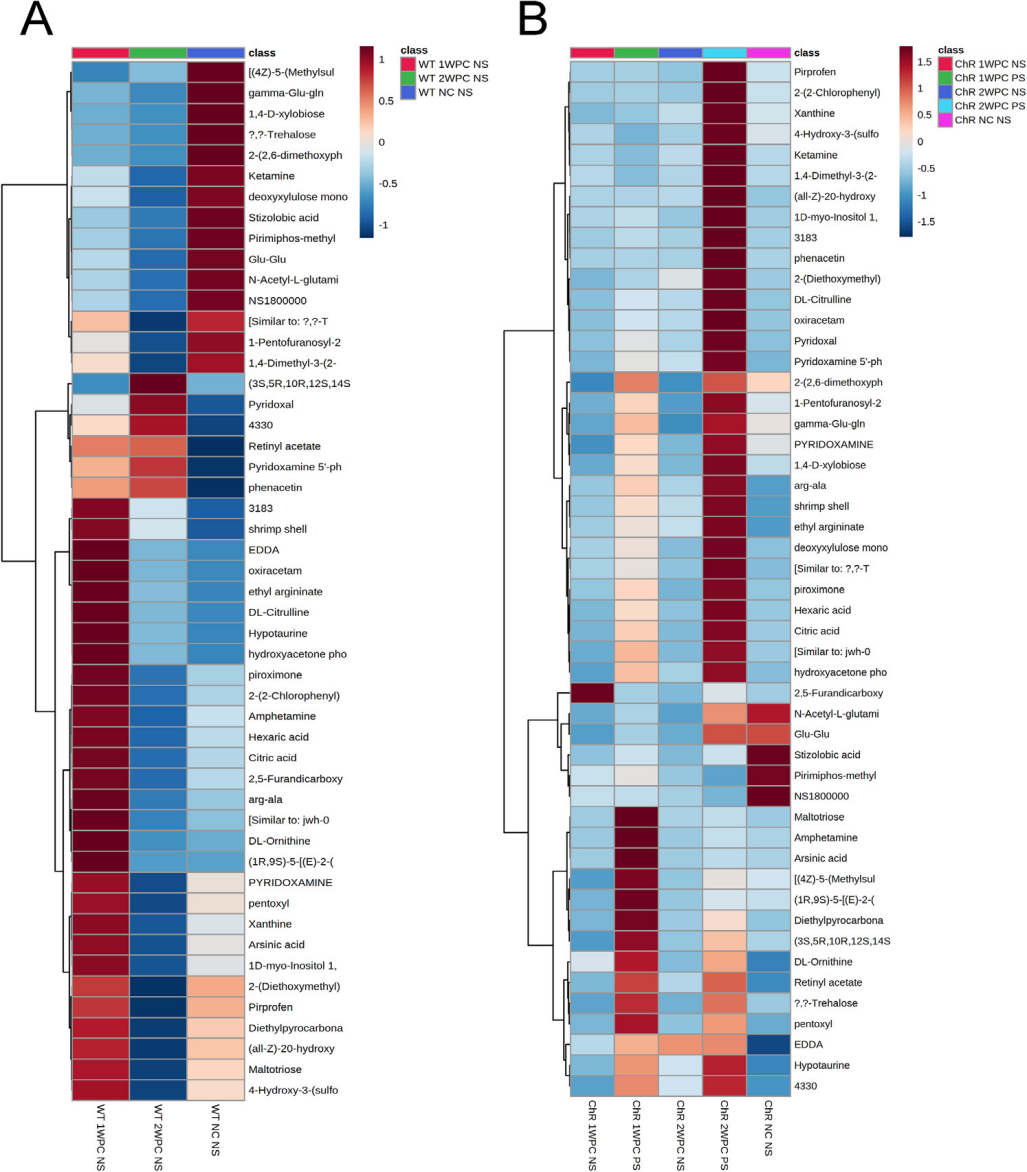
**Heatmap of the metabolite concentration changes in A) wild-type and B) channelrhodopsin mice.** Top 50 species are presented.

Raw scans were taken from a Q Exactive^TM^ Orbitrap^TM^ Mass Spectrometer (Thermo Scientific^TM^) and were processed and analyzed with Compound Discoverer^TM^ 3.3 software. Data was normalized and quantified to protein amount for bioinformatics analysis using MetaboAnalyst 5.0. Multivariate statistical analysis was performed using principal component analysis (PCA), partial least squares discriminant analysis (PLS-DA), and hierarchical clustering heatmaps (Fig. 2, Fig. 3). One-way ANOVA and post hoc analysis identified five important metabolite features: pyridoxamine, pyridoxal, N-Acetyl-L-glutamic acid, maltotriose, and 1-Pentofuranosyl-2,4(1H,3H)-pyrimidinedione. The graphical summary of the normalized distribution for each of these metabolites can be seen in Fig. S1. All additional supplementary materials can be found online.

## 2 Experimental Design, Materials and Methods

### 2.1 Animals

Usage of mice in experimental procedures were performed in accordance with the Animal Care and Use Committee at the University of Miami under the approved protocol 20-098. The Thy1-ChR2-eYFP and C57BL/6J mice were procured from Jackson Laboratory (Stock No. 007615 and 000664, respectively).

Thy1-ChR2-eFYP transgenic mice express the light-activated ion channel, channelrhodopsin (ChR2), fused to enhanced yellow fluorescent protein (eYFP) under the control of Thy1 promoter. Cells expressing ChR2 can be activated by blue light.

The Thy1-ChR2-eYFP and C57BL/6J mice (each 3-months old) were anesthetized using an intraperitoneal injection of a Ketamine/Xylazine cocktail. Before optic nerve crush, eyes received topical anesthesia using 0.5% Proparacaine. A 1 mm peritomy in the superior-temporal conjunctiva was performed, the ocular muscles were separated, and the retrobulbar optic nerve was exposed. The optic nerves were subsequently crushed using Dumont #5 Forceps (Fine Science Tools, Foster City, CA, USA) at approximately 0.5-1 mm behind the globe without damaging retinal vessels. Mice of both genders (equal distribution) were randomly assigned into either the optogenetic stimulation group or the control group. The mice in the optogenetic stimulation group received blue light stimulation (∼470 nm) at 1 Hz frequency while the mice in the control group were kept in a normal 12 hour light/dark environment. To deliver pulsed blue light, 20 blue light LEDs were fixed on a special ion cage and the mouse housing cage was placed in the ion cage. The output power of each LED is 10 mW (LED supply, 296 Beanville Road, Randolph, VT 05060). The frequency of serially connected LEDs were controlled by a programmable digital cycle timer (Uctronics, Nanjing, China). The mice were kept in the light stimulated environment one or two weeks after crush according to the experimental design.

### 2.2 Metabolite extraction

Metabolite extraction from samples was carried out in a 4 °C refrigerated room. Samples were transferred from long-term storage in a −80 °C refrigerator to the 4 °C room and kept on dry ice until homogenization and extraction was complete. 50 µL of chilled MeOH: H_2_O (1:1) was added to the microfuge tube containing the sample. Tissues were minced with small scissors in solution for two minutes. Samples were then vortexed for 45 seconds and centrifuged at 14,000 rpm for 20 minutes at 4 °C (Beckman Microfuge 18) to generate a protein pellet. Supernatants formed were transferred to a newly labeled microfuge tube. Fifty microliters of chilled acetonitrile:acetone:MeOH (8:1:1) was added to the original microfuge tube and these steps were repeated. Supernatants were transferred to the newly labeled microfuge tube and then dried in a speed vacuum at room temperature. Two extraction blanks were prepared in the same manner as the biological samples. This included all reagents, dissection tools, and instruments used from start to finish.

### 2.3 Untargeted liquid chromatography and mass spectrometry

Dried samples were reconstituted immediately in 100 µL of HPLC-MS grade water. Twenty µL of internal standard (D-Glucose-1,2,3,4,5,6,6-d_7_) was added to each sample. Samples were sonicated in an ultrasonic water bath for 25 minutes followed by centrifugation at 14,000 rpm for 5 minutes at 4 °C (Beckman Microfuge 18).

Pooled quality controls (QCs) containing all compounds representative of a batch were run in separate HPLC-MS vials to account for reproducibility and analyte stability. Pooled QCs were created by taking 10 µL aliquots of each sample and combined into a new tube.

Samples were subjected to fractionation and detection using a Thermo Scientific^TM^ Vanquish^TM^ Horizon Binary UHPLC. An Accucore^TM^ Vanquish^TM^ C18+ Column (100 mm x 2.1 mm, 1.5 µm, Thermo Scientific) was used to separate compounds with a flow rate of 0.300 mL/min. Mobile Phase A consisted of acetonitrile with 0.1% formic acid (v/v). Mobile Phase B consisted of water with 0.1% formic acid (v/v). Column temperature was set to 40 °C and injection volume at 5 µL.

The samples were run using a Q Exactive^TM^ mass spectrometer coupled to a heated electrospray ionization (HESI) source. The spray voltage was set to 3.50 kV, capillary temperature to 256 °C, sheath gas to 48, aux gas to 11, sweep gas to 2, and S-Lens RF Level to 50.0. The mass range was set to 67 – 1000 m/z, resolution 140,000 for full scan and 35,000 for ddMS^2^. AGC target was set to 3e6 for full scan and 2e5 for ddMS^2^. The max injection time (IT) was 200 seconds for full scan mode and 50 seconds for ddMS^2^. The number of microscans was 2, and normalized collision energy (NCE) was set to 20, 35, and 50. Samples were run in both positive and negative ion mode separately. The parameters for negative mode were the same except the spray voltage, which was set to 2.50 kV and the S-Lens RF level which was set to 60.0.

### 2.4 Metabolite identification and statistical analysis

Metabolites were identified from their Thermo.RAW scans using Compound Discoverer^TM^ 3.3 software. Extraction blanks were used to determine and correct for reagent effects, allow for the creation of exclusions lists, mark background components, and filter the background components from the results table in Compound Discoverer^TM^ 3.3. Pooled QCs were used for compound identification only.

Normalization to metabolite peak area was performed using the estimated protein amount created from the protein pellet during the extraction and analyzed using densitometry and ImageJ. Metabolites with duplicate peak areas were merged. Compounds identified in both positive and negative ionization mode were aligned. Metabolites and their respective normalized peak areas were uploaded to MetaboAnalyst. No data filtering, row-wise procedures, or data transformation was performed on MetaboAnalyst. Data scaling: range scaling was selected for further normalization.

For univariate analysis, a one-way ANOVA was performed (p-value threshold 0.05) followed by post hoc analysis (Fisher's LSD). Multivariate analysis included a principal component analysis (PCA) 2-D scores plot, partial least square-discriminant analysis (PLS-DA), and variable importance in projection (VIP) to identify the top 15 important features. Clustering analysis was performed in the form of a heatmap to show metabolite concentration changes (distance measured using Euclidean, Ward clustering algorithm, standardization using autoscale features).

## Ethics Statements

The study utilized animal models – mouse models encompassing both genders. All experiments were performed in compliance with the U.S. National Institute of Health Guide for the Care and Use of Laboratory Animals adhering to ARVO Statement for use of animals in vision research. Ethical guidelines of University of Miami under approved protocol 20-098. The sex of these mouse models is not known to influence or have an association with optic nerve regeneration.

## CRediT Author Statement

**Alexa Jauregui:** Formal analysis, Investigation, Data curation, Writing – original draft preparation, Writing - review & editing; **Yuan Liu:** Investigation, Writing – review & editing; **Richard K. Lee/Sanjoy K. Bhattacharya:** Conceptualization, Methodology, Supervision, Investigation, Writing – review & editing, Resources

## Declaration of Competing Interest

The authors declare that they have no known competing financial interests or personal relationships influencing the work reported in this paper.

## Data Availability

ST002111 (Original data) (Metabolomics Workbench).
